# Description of the Lubricant Behavior Based on the Theory of Elasto-Viscoplastic

**DOI:** 10.3390/ma18061360

**Published:** 2025-03-19

**Authors:** Yuriy O. Nosov, Anna A. Kamenskikh, Anastasia P. Bogdanova

**Affiliations:** Department of Computational Mathematics, Mechanics and Biomechanics, Perm National Research Polytechnic University, 614990 Perm, Russia; ura.4132@yandex.ru (Y.O.N.); anstasia_pankova@mail.ru (A.P.B.)

**Keywords:** numerical algorithm, mathematical model, Anand model, lubricant, thermophysical properties, computer-aided design systems

## Abstract

The aim of the work is to provide a mathematical description of the lubricant’s behavior model used in sliding bearings of bridge structures. It was previously established that the Maxwell model does not correctly describe the lubricant’s behavior in a wide range of temperatures and deformation rates. The lubricant model should take into account not only viscosity but also plasticity. The Anand model, which was adapted by introducing temperature dependencies for a number of material parameters, was chosen to describe the lubricant behavior. The functionality of the previously created procedure for identifying material properties was also expanded on the modified Anand model. This made it possible to obtain a lubricant mathematical model with an error of less than 5% in the operating temperature range from −40 to +80 °C. The study included a description of the behavior model for two lubricants: CIATIM-221 and CIATIM-221F. CIATIM-221F differs from CIATIM-221 by including superfine particles of polytetrafluoroethylene (PTFE) to improve properties. The study confirmed that the modified Anand model allows describing the material behavior more accurately than the Maxwell model. It was found that the samples behave as a solid over the entire temperature range (from −40 to +80 °C). A comparative analysis of the thermal behavior of CIATIM-221 and CIATIM-221F was performed.

## 1. Introduction

### 1.1. Research Objectives

The research objective is the description of the grease elasto-viscoplastic behavior based on the Anand model by means of multi-parameter optimization using a numerical procedure for identifying material constants based on experimental data. In the first stage of the research, a series of experiments were conducted to determine the thermophysical properties for greases over a range of operating temperatures (from −40 to +80 °C) and exposure frequencies (0.01 to 100 Hz). Further, the elasto-viscoplastic behavior of grease is described using the extended Anand model. Hypotheses about the temperature dependence of the parameters are formulated. The following stage is the expansion of the functional of numerical procedures to identify the mathematical model, which was developed earlier [1]. The final stage is to obtain the parameters of the equations for describing the behavior of materials, which allow obtaining the minimum error between experimental and numerical data (not more than 5%).

### 1.2. Problem Context

The increase in the rate of territory urbanization places new demands on transport systems in general and load-bearing elements in particular [1,2]. The issues of stability and strength of transport systems elements, especially bridge structures, are relevant [3,4,5]. This is due to the increase in loads from traffic flow and the expansion of highway construction in areas with difficult temperature conditions. Measures set for monitoring and predicting the behavior of critical components, their elements, and the materials from which they are made are actively developing [6,7,8,9]. Research is aimed at obtaining data on the structure’s behavior with an assessment of the ensuring safety possibility, increasing the load capacity, increasing the elements’ lifespan, etc. This research area is mainly related to the numerical analogs creation of structures and materials from which they are made [3,4]. These are metallic and non-metallic materials, including lubricants, which ensure stable operation of structures.

The bridge’s bearings are transport systems critical elements [10,11]. The bearings perceive load complexes from the bridge span and external climatic and man-made influences. It is the sliding bearings that allow bridge structures to perform the functions assigned to them. This occurs due to the redistribution of loads on the support. A number of research areas aimed at modifying the bearing designs can be identified: geometric configuration [12], materials of the elements [13], the position of the polymer sliding layers [3], etc. Engineering solutions are most often tested within the framework of numerical modeling. This leads to the need to build models of material behavior.

**Figure 1:**
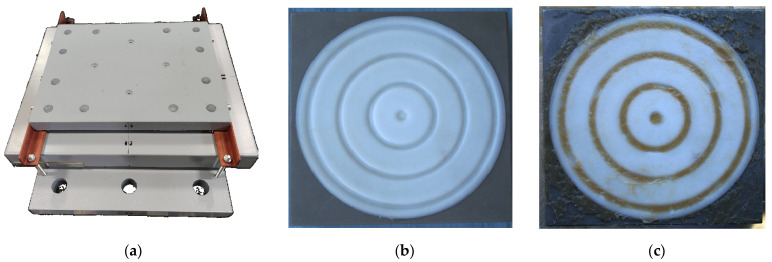
Spherical bridge bearing: (**a**) is assembled structure; (**b**) is polymer layer with cylindrical grooves without grease; (**c**) is polymer layer with cylindrical grooves filled grease.

Spherical sliding bearings are widely used in bridge construction [4,10]. The spherical sliding bearings contact unit includes a spherical steel balancer; lower steel plate; flat and spherical polymer sliding layers with technological recesses filled with grease (Figure 1).

One variant for the recesses geometric configuration for lubricant is presented in Figure 1. These are annular grooves. Other grease recess geometries also exist.

The bearings are designed for long periods of operation and perceive constantly changing cyclical and dynamic loads [14,15]. The effective operation of the structure depends on sliding layer materials: polymer/composite and grease [16,17,18]. The environment also influences the structure’s performance, especially temperature conditions and their changes [19,20,21]. The bearing numerical analogs are needed for analysis and predictive analytics of structure performance under different temperature and force conditions. The description of sliding layer materials within the framework of elasticity theory does not allow obtaining data close to the real structure behavior. Construction of nonlinear models of material behavior is required to minimize the deviation of the numerical simulation results. The mathematical description of polymer/composite and grease should take into account the influence of temperature and strain rate. Viscoelasticity theories and elasto-viscoplasticity theory can be applied to describe the material’s behavior.

### 1.3. Problem Description

This study is aimed at greases nonlinear behavior descriptions, which are widely used in the bridge industry, including in bridge sliding bearings [22,23]. Such lubricants are used in other industries: mechanical engineering [24,25,26], aerospace [27,28], biomedicine [29], etc. The constructed models can also find application in predicting the operation of contact units in other branches of science and technology.

The lubricants can significantly increase the durability of the structures: maintaining the required temperature, reducing frictional effects, damping the interaction between matting surfaces, etc. Lubricants are divided into liquid [30], solid [31], paste-like (grease) [32], etc. Liquid lubricants are often used in engines, transmissions, metalworking, hydraulic systems, and other mechanisms [25,33]. Solid lubricants [34,35,36] are used in highly loaded mechanisms: rolling bearings, gears, etc. The grease is widely used in sliding bearings [22,23].

Several lines of research are being considered to increase the service life of plain bearings.

The first direction of research is related to the bearings geometric configuration. This is mainly reflected in the sliding layer geometry to ensure stable lubrication of the contact surfaces. Changing the geometry is aimed at improving the lubricating characteristics [37]. Efficient lubrication of the mating surfaces minimizes the risk of metal-to-metal contact. This can lead to structural failure. A similar study was conducted by our group previously. It was aimed at increasing the grease amount by sliding layer geometry changing [22].

The second area of research is changing the lubricant composition. Tomanik et al. [38] examined the effect of different graphene-based additives on lubricants. The inclusion of various additives in the lubricant composition can also improve the properties [39]. Adding polymer inclusions to the lubricant composition is also an option modification [40]. But a comparative analysis of the lubricants behavior under conditions close to operational ones is required to assess the effect.

The authors [41] draw attention to the importance of studying the rheological characteristics of greases. The paper presents an experimental study of several greases used in wind power engineering over a wide range of operating temperatures. Han Peng et al. found that the effects of characteristics such as temperature, shear rate, viscosity, etc., influence the contact parameters of the system. This makes it possible to tailor a particular grease to specific operating modes of the structure.

Three directions of research related to lubricants exist at present:

1. Study of tribological, thermophysical, electromechanical and other grease properties.
2. Experimental study of the greases behavior under thermal and force influence.
3. Mathematical description of grease behavior for possible use in computer aided design (CAD) systems.

This study engages all three of the outlined research areas.

The creation of numerical analogs of structures based on data on the behavior of materials is also relevant [42,43]. CAD active development makes it possible to create design numerical analogs that differ slightly from the real object [44,45]: ANSYS Mechanical APDL (ANSYS Inc., Canonsburg, PA, USA); ABAQUS (ABAQUS Inc., Velizy-Villacoublay, France), etc. [46] notes the importance of creating numerical analogs of designs for the development of society as a whole and the transition to Industry 4.0.

### 1.4. About Mathematical Model

The application of the Anand mathematical model is widespread at present. This model is used to describe the behavior of many materials: metal alloys [44]; 3D printed structures [47,48], polymers [49], composites [50], rubbers [51], glasses [52], and other materials. Anssari-Benam notes that the large number of parameters of the Anand model complicates the identification and interpretation [53]. However, the model allows us to obtain higher-quality results on the materials and structures behavior that brings us closer to understanding the work of real objects. Despite the complexity of the model, many scientists are engaged in modifying the mathematical model to suit their own problems [54,55], in order to minimize the error between experimental studies and numerical data.

A thorough approach to the description of materials mathematical models is necessary to minimize the error between the real design and the numerical analog. This is the reason for a series of problems related to the selection of an adequate model of material behavior [56,57,58] and the search for the necessary system parameters to implement the tasks in modern CAD.

In [58], a description of the materials works like Maxwell solid using two mathematical models (Prony series and simplified Anand model). This work was carried out earlier by our research group.

The article found that:

- the mathematical model describes quite accurately the behavior of materials under temperature changes and at frequencies from 1 Hz to 100 Hz;
- there is an increase in the error relative to experimental data at frequencies from 0.01 to 1 Hz.

This effect may be associated with the choice of a fairly simple mathematical model to describe the greases behavior. For this reason, a decision was made to consider the possibility of describing the lubricants behavior in a wide range of operating temperatures and deformation rates using more complex mathematical models, such as the Anand model.

The article presents a study related to the description of the lubricant behavior model within the framework of the elasto-viscoplastic theory. Modification of the Annad model taking into account the dependence of a material constant’s number on temperature is implemented. Descriptions of the material behavior model are based on experimental data obtained over a wide range of temperatures (from −40 to +80 °C) and strain rates (from 0.01 to 100 Hz).

Our objective was to obtain a model that describes well the grease behavior within the framework of solid mechanics in a wide temperature range. The model will allow us to study the sliding bearing behavior of a bridge structure under cyclic and dynamic loads. A new grease behavior model based on the Anand model was obtained and tested on two materials.

There was also an interest in assessing the effect on the deformation behavior of grease of adding ultra-dispersive polytetrafluoroethylene (PTFE) or nano-PTFE. That is also presented in the article. New experimental data on the grease behavior is obtained. Thermomechanical properties are built on dependences of greases on temperature and deformation rate. The properties are described by a mathematical model. The data will find wide application in predictive analysis of the friction unit’s operation in dynamics, for example, spherical bearings.

## 2. Materials and Methods

### 2.1. Materials

A number of greases developed by the Central Institute of Aviation Fuels and Oils (CIATIM, Moscow, Russia) were selected as study objects: CIATIM-221 and CIATIM-221F.

CIATIM-221 is a thermostable, waterproof, hygroscopic, wearproof, multi-purpose grease. The grease structure is homogeneous. Color is from light yellow to light brown. Polysiloxane fluid is the grease base. Stearate acetate calcium is a thickener. The grease is inert and chemically resistant to polymers and rubber. The grease contains the additive diphenylamine.

CIATIM-221F is a modification of the CIATIM-221. The nano-PTFE is added in the grease composition. The grease has improved extreme pressure and antiwear properties. A tribo-film forms on the mating surfaces when grease of this type is used. This effect occurs due to the nano-PTFE addition. The grease has improved heat thermostability, mechanical stability, and nonhygroscopic properties.

Both greases are compatible with plastics and polymers and work well by mating metal to metal and metal to rubber.

**Figure 2:**
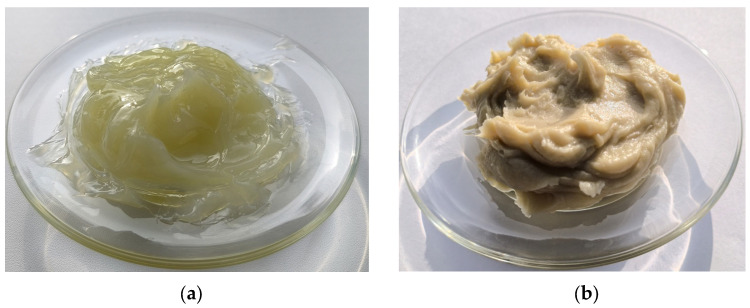
Grease samples: (**a**) is CIATIM-221; (**b**) is CIATIM-221F.

The CIATIM-221 and CIATIM-221F samples are shown in Figure 2.

There are different ways to obtain greases with nano-PTFE. Dispersion of PTFE in aqueous or oil medium [59,60]. The resulting suspension is adding to the grease composition with constant mixing. The grease is stabilized by additives: surfactants, block copolymers, and/or polymers. Various technological processes are used to obtain a homogeneous grease structure [61]: ultrasonication, homogenizing, magnetic force agitation, probe sonication, ball milling, and high shear mixing. The lubricant-PTFE concentrate preparation, which is composed into the grease, is an alternative production process [62]. The grease-PTFE composition thermal heating to the polymer melting temperature is also encountered in the technological process [59]. But the exact technological process for the CIATIM-221F production is not presented in open sources, as it is considered a trade secret.

### 2.2. Experimental Study

**Figure 3:**
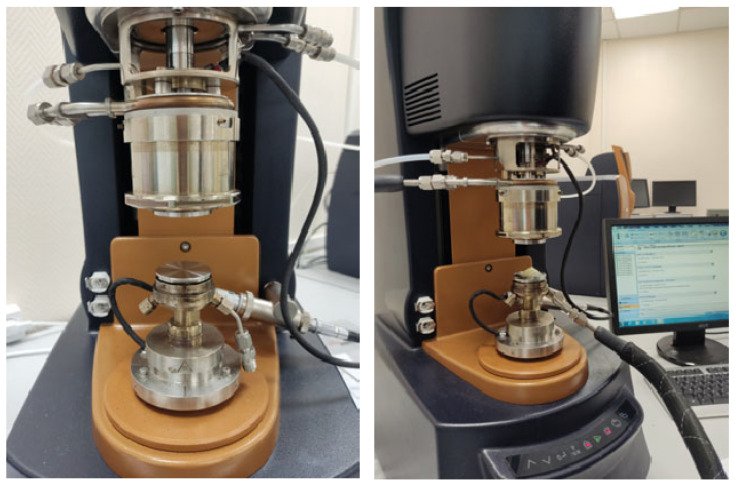
Experimental research on the Discovery HR2.

Dynamic modal analysis (DMA) is widely used as one of the methods for experimental determination of thermophysical characteristics. The paper performed an experimental study on a Discovery Hybrid Rheometer 2 (DHR 2), which allows the determination of rheological characteristics of materials for a wide range of temperatures [58]. The experimental setup on the Discovery HR2 is shown in Figure 3.

The torque M and the displacement angle φ are the output data of the DHR 2. The values of true stresses (1) and true strains (2) are calculated using the following formulas:

(1) $$\tau \;=\;K_{\tau }\cdot M,$$

(2) $$\gamma \;=\;K_{\gamma }\cdot \varphi ,$$

where $K_{\tau }$ and $K_{\gamma }$ are stress and strain constants, respectively, depending on the mating surfaces geometry, τ is tangential stress, γ is shear strain.

**Figure 4:**
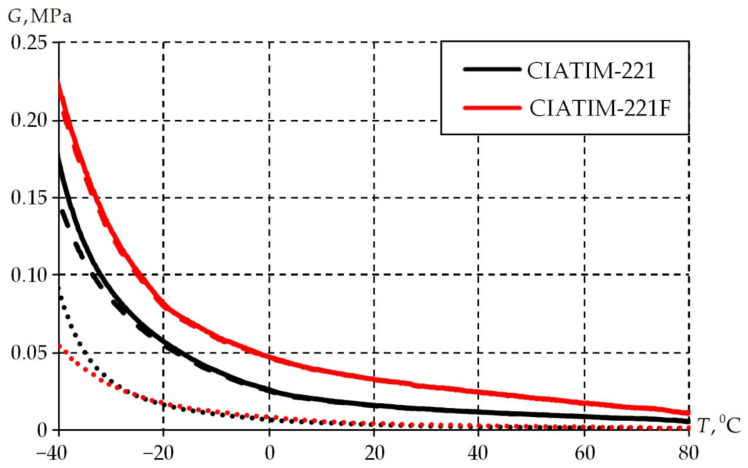
Dependence of shear modulus components on temperature: $G^{*}$ is solid; G′ is dashed; G″ is point.

The previously tested procedure [58] will be applied for the determination of viscoelasticity parameters. The first step is to determine the possibility of its use. The following conditions have to be met for the material: It has a constant aggregate state over the entire range of temperatures considered (from −40 to +80 °C at a heating rate of 2 deg/min); it is elastic or gel. For this purpose, the article analyzed the aggregate state of the material by the dependence of the complex shear modulus $G^{*}$, storage modulus G′ and loss modulus G″ on temperature (Figure 4).

A material is solid or gel if, over the entire temperature range, the loss modulus is lower than the storage modulus [58,63]. This distribution of experimental data is observed in Figure 3. Consequently, the application of the procedure for the calculation of viscoelastic parameters is reasonable.

### 2.3. Mathematical Model

Description of lubricants within the model of a series connection of an elastic spring and a viscous element (Maxwell body) is widely used [64,65,66,67]. Prony series and a simplified Anand model are used to describe grease as a Maxwell body [68]. This fundamental model is simple in description in relation to the well-known models of Kelvin, Voigt, etc. And it is suitable for a description of material behavior within the first approximation.

However, the research team previously found the following limitations [58]: Using Maxwell’s equations, the model works only at high strain rates; the simplified Anand model is only suitable for frequencies greater than 1 Hz. A significant error is observed at small frequencies (0 to 1 Hz). Therefore, this paper considers the full Anand model to describe the behavior of grease.

The modified Anand model for describing the greases behavior will allow you to analyze the sliding bearings work at a wide range of operating temperatures. The study results will find wide application in the bridge bearings design, especially in regions with difficult climatic conditions [69,70,71].

The basic equation has the form:

(3) $${\dot{\gamma }}_{v}=Ae^{-U/RT}{\left[\sinh\left(\xi \tau /S\right)\right]}^{1/m},$$

It includes an evolutionary equation:

(4) $$\dot{S}=\{h_{0}{\left(\left|B\right|\right)}^{a}B/\left|B\right|\}{\dot{\gamma }}_{v},$$

where $B=1-S/S^{*}$; $S^{*}=S_{1}{\left[{\dot{\gamma }}_{v}e^{U/RT}/A\right]}^{n}$, A is pre-exponential factor, ξ is dimensionless scalar constant, m is strain-rate sensitivity of stress, τ is tangential stress, S is deformation resistance, $S_{0}$ is initial deformation resistance; $S^{*}$ is saturation value of the hardening function, $h_{0}$ is hardening/softening constant, R is universal constant gas, U is activation energy, T is absolute temperature, n is sample saturation as a function of shear rate, a is strain rate sensitivity of hardening/softening. The majority of modern engineering packages ANSYS Mechanical APDL 2021R2 (Livermore, CA, USA) (for FEM analysis) require a vector of constants to be specified to describe the material behavior by the Anand model:

(5) $$\bar{x}=\left\{S_{0};A;\;\;U/R;\;\xi ;\;m;\;h_{0};S_{1};n;a\right\},$$

According to authors [50,51,52] and experimental data, a hypothesis of dependence of initial strain resistance, and strain-rate sensitivity of the material on temperature is possible. Here let present these parameters in the form of (6) and (7), respectively, to confirm this hypothesis:

(6) $$S_{0}\left(T\right)=C_{1}\left(1+C_{2}e^{C_{3}/T}\right)\left(1+C_{4}e^{C_{5}/T}\right),$$

(7) $$m\left(T\right)=C_{6}\left(1+C_{7}e^{C_{8}/T}\right)\left(1+C_{9}e^{C_{10}/T}\right),$$

where $C_{1}-C_{5}$, $C_{6}-C_{10}$ are coefficients for describing the behavior of the initial strain resistance and strain-rate sensitivity depending on the temperature, respectively. The vector of unknowns is formed by substituting Equations (6) and (7) into the vector of coefficients (5) $\bar{x}=\left\{A;\;\;U/R;\;h_{0};S_{1};n;a;\;C_{i},\;\;i=\bar{1,10}\right\}$.

### 2.4. Anand Model Identification Procedure

**Figure 5:**
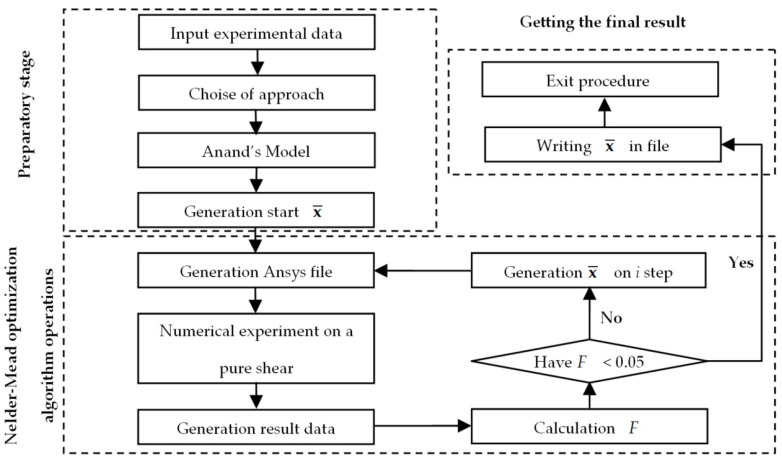
Simplified scheme of the numerical identification procedure.

Figure 5 illustrates the numerical procedure for identifying the mathematical model of grease behavior. The algorithm is implemented by Python 3.12 and ANSYS Mechanical APDL 2021R2. It allows replicating the experimental research. The parameters required for the model are searched for using Nelder–Mead multi-parameter optimization. This process is realized by iterative substitution of unknown parameters until a given error is obtained.

A previously developed procedure for numerical identification of materials viscoelastic properties was tested on lubricants [58] and polymers [72]. An assessment of the possibility of using the procedure to describe the photopolymer material Envisiontec SI500 behavior, used in additive manufacturing and 3D printing, was performed [73,74]. The effectiveness of the numerical identification procedure was confirmed by the agreement of the Envisiontec SI500 material model obtained using it with the results of Shumkov et al. [74].

The numerical procedure consists of several steps:

(1) Experimental data are loaded. The type of mathematical model is selected. The initial vector of unknowns is formed. Section 2.3 describes the mathematical equations of the algorithm detail.
(2) A numerical experiment is realized.

**Figure 6:**
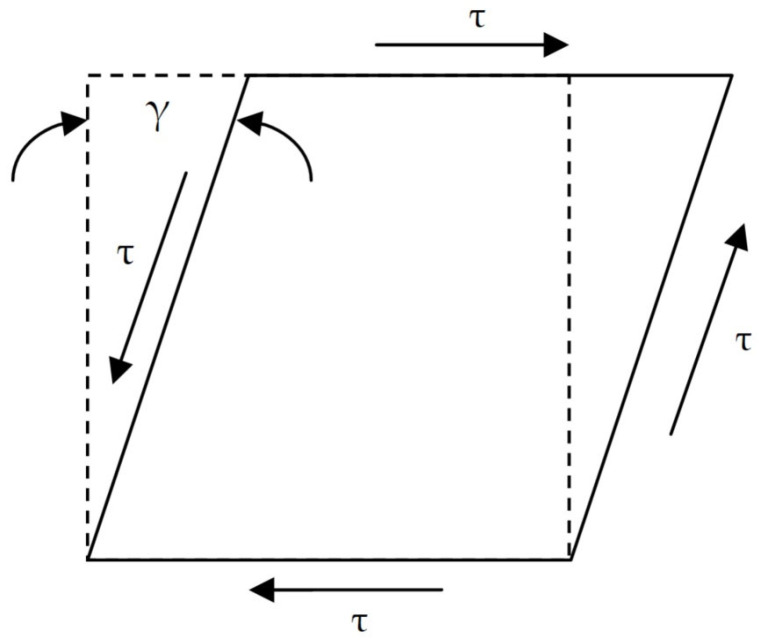
Numerical simulation of the shear stress.

Two variants of numerical simulations of the shear stress were implemented (Figure 6):

- the changes in the frequency from 0.01 to 100 Hz at constant temperatures;
- the temperature change from −40 to +80 °C in a step of 2° per min, with a constant frequency (1 Hz).

The search for the unknown coefficients $\bar{x}$ occurs as step-by-step procedure. The residual function has the form (8). The procedure is stopped when the residual is less than 1%:

(8) $$F=\left({\bar{\tau }}^{\exp}-{\bar{\tau }}^{\mathrm{num}}\left(\bar{x}\right)\right)/{\bar{\tau }}^{\exp}\to \min,$$

Nelder–Mead multi-parameter optimization is selected for the task implementation.

(3) The work of the numerical procedure is completed. The file with the found parameters of the vector of unknowns is formed.

The presented approach can be applied to a wide range of materials. Previously, the applicability of the first algorithm version was evaluated to describe the polymer behavior [51].

The proposed model allows describing the grease thermomechanics only within the framework of the deformable solid mechanics. The model is applicable when the grease operates under uniform compression conditions. This type of grease deformation is realized in sliding bearings of bridge structures.

## 3. Results

### 3.1. Results of the Experimental Study

**Figure 7:**
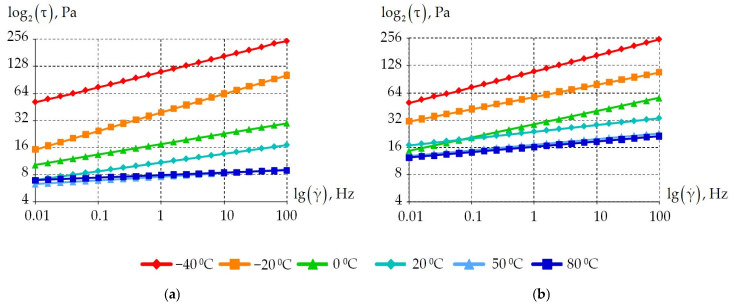
Dependence of the samples tangent stress on the frequency at different temperatures: (**a**) is CIATIM-221; (**b**) is CIATIM-221F.

The first stage of the study is to analyze the dependence of the grease stress state on frequency stimulation. For this purpose, a frequency in the range from 0.01 to 100 Hz was applied to the samples. Figure 7 shows the dependence of tangential stresses on frequency at different temperatures.

The pattern of the dependence of tangential stresses on frequency is linear. For all the greases (CIATIM-221, CIATIM-221F) considered, tangential stresses increase with increasing frequency. It can be noted that the introduction of PTFE into the lubricant composition affects tangential stresses at temperatures of −20 °C and above. The nano-PTFE addition to the grease composition increases the material’s resistance to frequency impact. This was obtained from the experiment. Similar results are observed in the other authors’ works [75,76,77]. This is an important factor for increasing the durability of highly loaded friction units.

**Figure 8:**
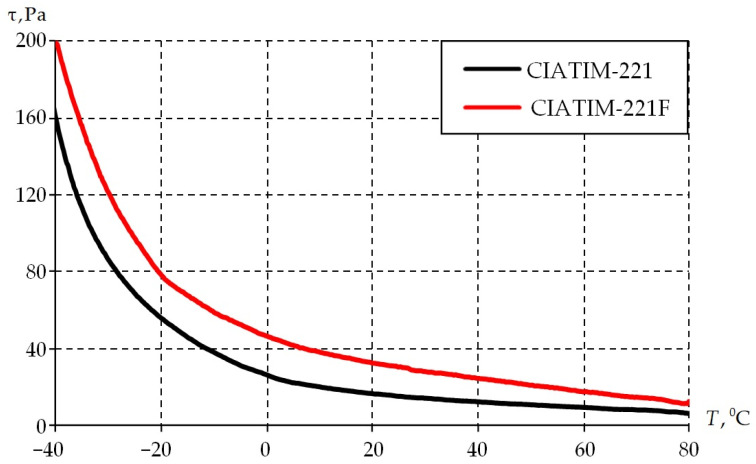
Dependence of the samples tangent stress on the temperatures at different frequency.

The second stage consists of studying the dependence of material properties on temperature. The sample is cooled from +80 to −40 °C in steps of 2° per min while being subjected to cyclic deformation with a frequency of 1 Hz. The results are presented in Figure 8.

The physical and mechanical properties of the grease improved with the introduction of PTFE into its composition. It is observed that it is necessary to increase the torque value with decreasing temperature to keep the magnitude of sample deformation at the same level (0.1%). The CIATIM-221F physical and mechanical characteristics are, on average, 20% higher. This increases the stability of tribological and rheological characteristics during the structure deformation.

### 3.2. Results of the Anand Mathematical Model Identification

Identification of Equations (3), (4), (6), and (7) parameters was performed within the developed numerical procedure. The initial vector of unknowns is the same for both greases (CIATIM-221 and CIATIM-221F). The initial and final values of the unknowns independent of temperature are presented in Table 1.

Sample saturation as a function of shear rate and strain-rate sensitivity differ by no more than 6% for each material. Significant differences are observed in the pre-exponential factor A (about 65%). The material hardening/softening constant $h_{0}$ has maximum differences, namely: $h_{0}$ of CIATIM-221F is approximately 1.5 times greater than at CIATIM-221.

**Figure 9:**
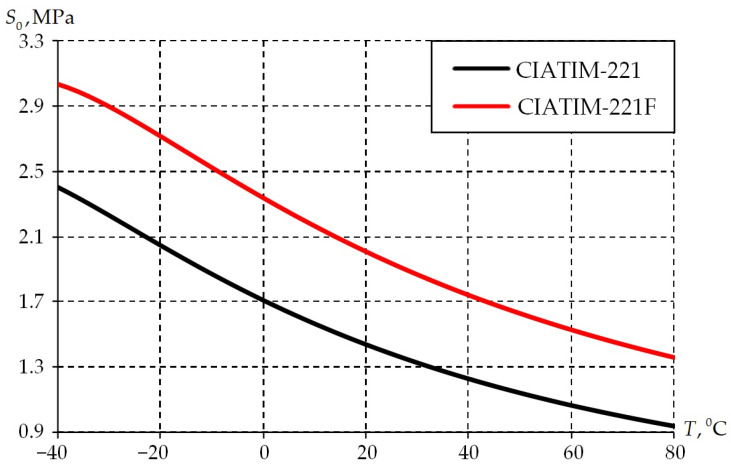
Temperature dependence of the initial deformation resistance.

**Figure 10:**
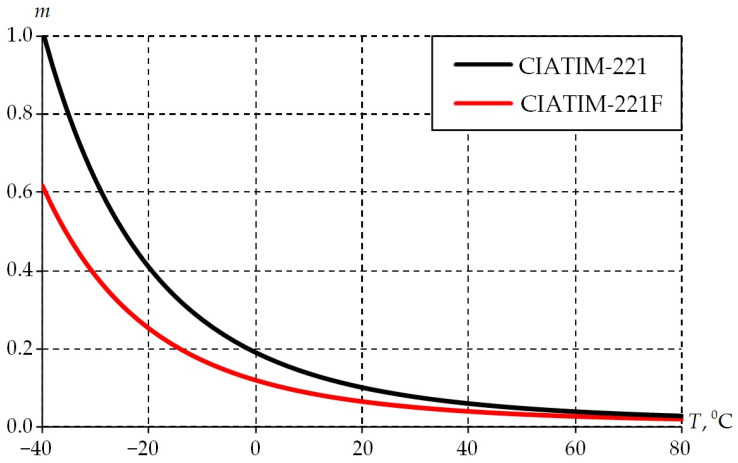
Temperature dependence of strain-rate sensitivity of stress.

The temperature dependence of the grease parameters was obtained: the initial deformation resistance (Table 2, Figure 9); strain-rate sensitivity (Table 3, Figure 10).

The distribution of the initial deformation resistance $S_{0}$ is illustrated in Figure 9.

The temperature dependences of the initial strain resistance are qualitatively similar. The initial deformation resistance decreases over the entire operating temperature range. The dependence of the initial deformation resistance on temperature is nonlinear. After further increase in temperature, there is a decrease in the values according to a close to linear law. The initial deformation resistance of the CIATIM-221F is almost 20% times higher than CIATIM-221. This is due to the nano-PTFE addition to the grease composition.

Figure 10 shows the distributions of strain rate sensitivity of stress m over the entire temperature range.

The temperature dependence of strain rate sensitivity of stress has a similar distribution pattern as the temperature dependence of the activation energy. However, in this case, the highest value prevails in the CIATIM-221 in the temperature range from −40 °C to 5 °C. The deformation rate has a significant effect on the CIATIM-221 behavior. The nano-PTFE addition in the composition CIATIM-221F makes the grease less sensitive to deformation rates.

The identification procedure was performed on a personal computer equipped with an Intel Core i9-10900F, 128 GB RAM, and 4 TB SSD. The procedure finds solutions of a given accuracy from 2 to 30 days. The convergence rate of the solution strongly depends on the initial vector of unknown parameters. It can be increased by implementing the problem on modern server platforms and clusters. In further research, it is planned to evaluate the stability of the numerical procedure.

In addition, it is planned to compare experimental data and numerical calculation implementing the full Anand model to describe the grease behavior.

### 3.3. Frequency and Temperature Dependence of Physical and Mechanical Characteristics

The identification procedure of unknown material parameters allowed obtaining coefficients for describing greases by the Anand model. Numerical simulation of the pure shear experiment was performed in ANSYS. The paper implemented a custom script using APDL to automate a series of numerical calculations on the temperature and deformation parameters of the system. In addition, the algorithms include automatic processing of the numerical solutions.

**Figure 11:**
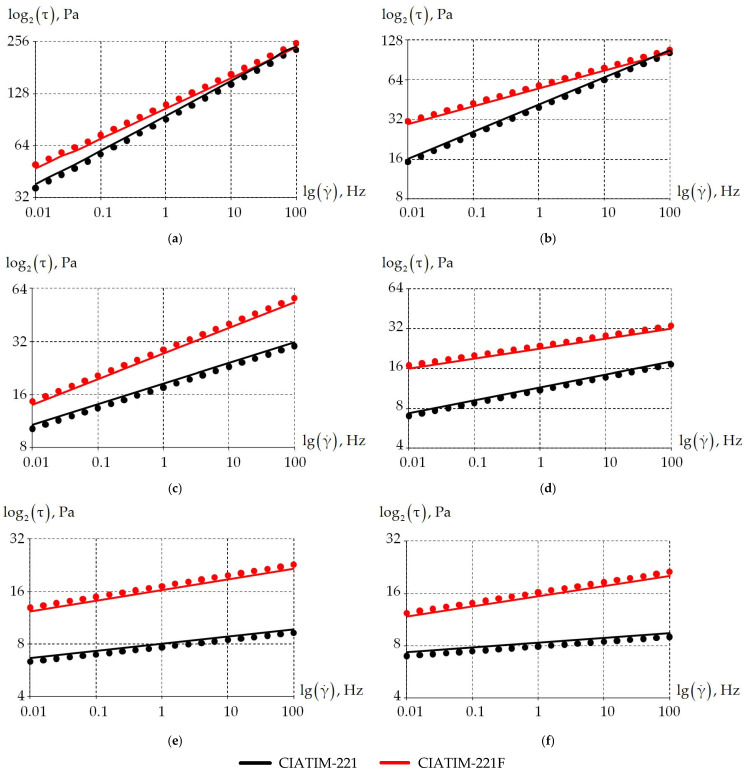
The frequency-shear dependence of the tangential stress in the temperature range: (**a**) is −40 °C; (**b**) is −20 °C; (**c**) is 0 °C; (**d**) is 20 °C; (**e**) is 50 °C; (**f**) is 80 °C; dots is experiment; solid is numerical calculation.

Figure 11 demonstrates the frequency-shear dependence of the tangential stress values at specific temperatures in the range from −40 °C to 80 °C.

The numerical calculation qualitatively and quantitatively corresponds to the experimental data over the entire temperature range. The total error at the points under study does not exceed 1%.

**Figure 12:**
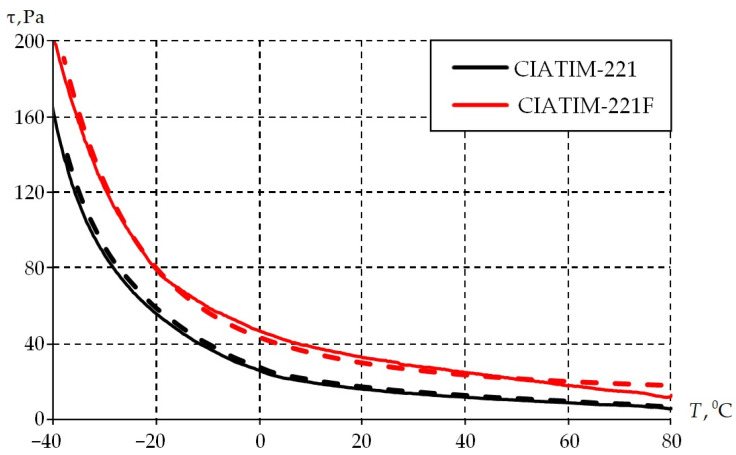
The temperature dependence of the tangential stresses: solid is experiment; dashed is numerical calculation.

The temperature dependence of the tangential stresses is shown in Figure 12. The numerical model was exposed to temperatures from +80 to −40 °C in steps of 2 °C per minute. The frequency is constant and equal to 1 Hz. The conditions of numerical calculations completely repeat the experiment.

Numerical calculations qualitatively and quantitatively correspond to the experimental data. The difference between the results of the numerical model and the experimental data is less than 5%. The full Anand model allows us to describe the viscoelastic grease behavior. It can be used for a wide class of problems on predicting the behavior of materials and structures over a wide range of temperatures under dynamic loads.

## 4. Discussion

### 4.1. Limitation Statement

The paper investigated the applicability of the Anand model to describe the grease behavior. The following constraints are established as part of the study:

- considered a temperature range of −40 °C to +80 °C, with −60 °C and +150 °C being the minimum and maximum temperature values for the feasibility of the grease, respectively;
- there is no possibility to carry out the experiment by means of non-contact impact on the sample using DWS technology [78];
- there is no open database of test materials and information on initial vectors for Equation (5), which leads to increased time for identification of the mathematical model of material behavior;
- there are no separate experimental studies on the determination of the rheology and creep of greases in open sources.

In the future, the researchers will have to complete a number of tasks:

- conducting material studies at near-critical temperatures (−60 °C and +150 °C);
- enriching the experimental database to reduce the time required to identify mathematical models of greases for future studies;
- conducting additional experiments on the rheology and creep of materials;
- conducting experiments by means of non-contact impact on the sample using DWS technology;
- extending the applicability of the mathematical model to describe the behavior of polymers and composites.

### 4.2. About the Mathematical Models

These studies allowed us to obtain a parameter change range that significantly accelerates the mathematical description of a lubricant class, which includes CIATIM-221 and CIATIM-221F.

As well, an important result of this work is the expansion of the procedure’s functionality by describing the full and modified Anand model, which can be applied to a wide range of materials: polymers, composites, greases, etc. It was chosen as a first approximation as one of the popular models.

A fairly large set of complex models that allow one to obtain a description of a lubricant as a viscoelastic or elasto-viscoplastic body, considering various properties [79,80,81,82,83,84].

Many researchers consider the behavior of lubricants (in particular grease) under certain conditions. There is a growing tendency to consider these materials using nonlinear models of viscoelastic/elasto-viscoplastic behavior. As noted by the authors [85,86,87], most of the existing equations do not allow for performing modeling of grease behavior considering all properties, yield modes, etc. As a consequence, there is a great variety of models and their modifications, which are used to take into account dripping [88], thixotropy [82], cavitation [79], etc.

Most researchers consider lubricants as a liquid [84,89,90]. For this purpose, such models are used as the Carreau model [89], its modification—the Carreau–Yasuda model [89]; the Herschel–Bulkley model [39,90]; etc. The Carreau–Jasuda model is used by the authors [88] to describe the rheological properties of the tested greases. The Herschel-Bulkley model is in turn applied to describe lubricant as a single-phase liquid with different rheological properties [41,90]. Also, researchers [87] compared the description of behavior by rheological models with experimental data for two types of greases. They obtained a sufficiently good description using the Herschel–Bulkley model.

However, depending on certain factors, lubricants and greases can be considered as solid or gel. In our case, such consideration is possible because over the entire temperature range the loss modulus is lower than the storage modulus. Models such as Anand, Prony, Bingham, and the Oldroyd-B model [79], etc., in [87] with various mathematical modifications are actively applied to describe greases. There are solutions that describe the behavior of grease by symbiosis of Maxwell’s equation with other equations. Thus, [91] uses Maxwell’s equations with thermodynamic equations by replacing the classical work of compression PdV by the work of grease shearing Vτdγ. The authors of [86] present a number of existing solutions (Maxwell–Wichert model, Kelvin–Foigt model, etc.) for describing the nonlinear complex lubricant behavior in the framework of contact problems.

Most researchers are guided by both basic properties (rheological, physical-mechanical, etc.) and particular properties of grease when describing their behavior. Thus, in their studies, Marek Wozniak et al. [88] consider the influence of the dropping temperature on the behavior of grease at different temperatures.

Thixotropy is a property in which the material relaxes over time when the load is removed, and the viscosity decreases when the load is applied. There are different approaches to describing thixotropy, which have their limitations and advantages. For example, the authors of [83] provide a description of several model types for elasto-viscoplastic thixotropic modeling. They note that the Bingham model is quite often used to describe the elasto-viscoplastic behavior of materials with thixotropic properties. In addition, they consider the option of combining models describing solids and liquids, which in general is an interesting approach to modeling complex lubricant behavior.

In our case, for the greases considered in this paper (CIATIM-221/221F), no experiments have been performed to investigate relaxation and reveal the properties of thixotropy. This may be one of the stages of research on the nonlinear behavior of greases.

An important result of this work is the expansion of the procedure’s functionality by describing the full and modified Anand model, which can be applied to a wide range of materials: polymers, composites, greases, etc. This statement is true if the material is considered within the framework of solid mechanics. But it is important to conduct experimental research of greases over the entire range of operating temperatures. These studies also allowed us to obtain a parameter change range that significantly accelerates the mathematical description of a grease class, which includes CIATIM-221 and CIATIM-221F.

In the context of further research, we will consider the possibility of expanding the procedure for identifying properties to these models. This in turn will allow us to more fully evaluate the effectiveness of describing a lubricant using the modified Anand model. The main disadvantage of the modified Anand model is the large set of unknown parameters, which leads to significant costs of computational and time resources when implementing the task.

### 4.3. About Grease Composition

Changing the greases composition by nano-additives means allowing the improvement of the material’s tribology [92,93,94]. The influence of nano-additives on the greases rheology is also analyzed [93]. Positive effects on the material’s rheology are noted. This leads to an increase in the highly loaded friction units’ service life [95,96,97]. The effect of the nano-PTFE additive in grease is still not fully understood. Physical-mechanical, tribological, rheological, and other properties of grease after modification are being studied by scientists. The influence of the size and geometric parameters of PTFE particles on the performance characteristics of greases is analyzed [98]. The study showed that 6 µm PTFE spherical particles can maximally improve the grease tribological and performance characteristics. In [99], it was determined that excessive nano-PTFE concentration leads to deterioration of grease tribological characteristics. The manufacturing process and the grease composition have a significant impact on the material behavior [95,96]. CIATIM-221F with nano-PTFE additive has improved properties in this study. The material decomposition was not observed. This indicates the composition stability. But the exact recipe and technological process data are not provided in open sources.

Commercial secrets of manufacturing companies hinder the possibility of obtaining a grease complete description from the technological process of creation to operation in real structures. The mathematical model behavior description of grease is the main goal of this study. The chemical composition and technological process of production have not been studied comprehensively due to the manufacturer’s trade secrets. However, there is a shortage of qualitative descriptions of grease nonlinear behavior [100,101]. This indicates the presented study’s importance for the industry’s development. The data are also relevant for predictive analysis of friction units within the framework of computer engineering.

## 5. Conclusions

The article carried out a series of experiments to determine the thermophysical properties of greases in a wide range of temperatures (from −40 to +80 °C) and shear rates (0.01 to 100 Hz). The hypothesis is proposed about the temperature dependence of the Anand model parameters. New data on the temperature dependence of the activation energy distribution, initial resistance, and shear rate sensitivity are obtained.

The numerical procedure has been created to determine the Anand model coefficients in the synergy of ANSYS Mechanical APDL and Python.

The following conclusions are drawn:

- The strength properties of greases increase with decreasing temperature.
- Consideration of the temperature effect on material behavior is necessary for qualitative and quantitative descriptions of thermophysical grease properties.

The article obtained the coefficients of the extended Anand model for describing the grease behavior. Temperature dependences are determined for some of its parameters. The error is less than 1% between experimental and numerical data when using the extended Anand model.

In the development of the work, it is planned to evaluate the possibility of using these algorithms to describe the performance of sliding layer polymers of bearing.

Analysis of grease chemical composition is planned as part of future research. Expansion of the grease range is also planned.

## 6. Patents

A state registration certificate of a computer program of the Russian Federation “Mathematical model identification of the Maxwell body viscoelastic behavior based on Prony series” No. 2023618695, registration date 27 April 2023. The authors are Anna A. Kamenskikh and Yuriy Nosov. The holder is Perm National Research Polytechnic University.

## Figures and Tables

**Table 1:** Values coefficients of Anand model for CIATIM-221 and CIATIM-221F.

Values	A , 1/s	U/R, K	ξ	$h_{0},\;\;\mathrm{MPa}$	$S_{1},\;\;\mathrm{MPa}$	n	a
Initial	1 × 10^−6^	1000	1 × 10^−2^	1 × 10^−1^	1 × 10^−1^	1	1
${\mathrm{Final}}^{\mathrm{CIATIM}-221}$	1.389 × 10^−3^	1839.31	9.825 × 10^−2^	162.62	4.705 × 10^−1^	2.813 × 10^−1^	2.954
${\mathrm{Final}}^{\mathrm{CIATIM}-221F}$	8.697 × 10^−4^	1662.98	5.067 × 10^−2^	201.06	8.617 × 10^−1^	2.142 × 10^−1^	4.277

**Table 2:** Values of initial deformation resistance coefficients for CIATIM-221 and CIATIM-221F.

Values	$C_{1},\;\mathrm{MPa}$	$C_{2}$	$C_{3},\;\;K$	$C_{4}$	$C_{5},\;\;K$
Initial	1 × 10^−6^	1	100	1	100
${\mathrm{Final}}^{\mathrm{CIATIM}-221}$	1.437 × 10^−1^	−6.021 × 10^−3^	966.53	3.824 × 10^−1^	983.29
${\mathrm{Final}}^{\mathrm{CIATIM}-221F}$	1.959 × 10^−1^	−6.766 × 10^−3^	1070.53	4.886 × 10^−1^	927.01

**Table 3:** Strain-rate sensitivity values for CIATIM-221 and CIATIM-221F.

Values	$C_{6}$	$C_{7}$	$C_{8},\;\;K$	$C_{9}$	$C_{10},\;\;K$
Initial	1	1	100	1	100
${\mathrm{Final}}^{\mathrm{CIATIM}-221}$	9.060 × 10^−3^	5.894 × 10^−3^	−688.582	9.221 × 10^−4^	2728.20
${\mathrm{Final}}^{\mathrm{CIATIM}-221F}$	6.750 × 10^−3^	7.140 × 10^−2^	23.397	7.728 × 10^−4^	2703.05

## Data Availability

The original contributions presented in this study are included in the article. Further inquiries can be directed to the corresponding author.
